# Challenges and adaptations of mental health services during the COVID-19 Pandemic in Uganda

**DOI:** 10.11604/pamj.2024.48.84.43031

**Published:** 2024-07-02

**Authors:** Anita Arinda, Kenneth Kalani, Emmanuel Mpamizo, Raymond Sebuliba, Vanessa Akinyange, Sarah Lofgren

**Affiliations:** 1 Department of Psychiatry, College of Health Sciences, Makerere University Kampala, Uganda,; 2 Ministry of Health, Kampala, Uganda,; 3 Department of Psychiatry, Gulu University, Gulu, Uganda,; 4 Infectious Disease Institute, Makerere University, Kampala, Uganda,; 5 Division of Infectious Diseases and International Medicine, University of Minnesota, Minneapolis, Minnesota, USA

**Keywords:** Mental health services, COVID-19, continuity of patient care, Uganda

## Abstract

**Introduction:**

Coronavirus disease (COVID-19) significantly impacted mental health and mental health services worldwide. We sought to explore the challenges faced by mental health services from the perspectives of service users, providers, and policymakers during the COVID-19 pandemic in Uganda and the strategies put in place to ensure the continuity of these services.

**Methods:**

qualitative data were collected using semi-structured interviews with eight mental health service users, four mental health workers, four hospital administrators, four district mental health focal persons, and two policymakers. The data were analyzed using thematic analysis.

**Results:**

the challenges reported by participants included human resource shortages, loss of space for service provision, low funding, drug shortages, changes in patient load, and lack of access to services due to restrictive measures. The adaptations included the use of innovative means like mobile phone technology, reorientation of health facility functioning to COVID-19 restrictions, using different methods to deliver medications, integration of mental health in general health services, and alternative financing for mental health services.

**Conclusion:**

the COVID-19 pandemic posed significant challenges to mental health service provision. Nevertheless, the health system responded by implementing various measures to ensure continuity of care. Further research is needed to evaluate the effectiveness and scalability of these innovations in the long term.

## Introduction

The COVID-19 pandemic, which began in December 2019, was declared a public health emergency by the World Health Organization (WHO) in March 2020 [1]. This prompted countries to implement strict measures such as social distancing, school closures, remote work, and travel restrictions. Uganda experienced two lockdowns, from March to June 2020 and June to August 2021, which disrupted health services [2].

The pandemic had direct and indirect impacts on mental health, resulting in increased demand for services [3]. Factors like the contagiousness of the virus, fear of infection, strict measures like lockdowns and physical distancing, and socio-economic disruptions negatively affected mental health [4,5]. This led to higher rates of mental distress and conditions like depression, anxiety, and substance use [6]. Studies conducted in Uganda reported high levels of psychological distress, depression, and anxiety among various groups including, the general population [7,8], students [9] and frontline workers [10]. Mental health systems faced challenges such as service disruption, limited access to care and medication, shortages of mental health professionals, and staff burnout [11-14]. The disruption of care for mental illness can be life-threatening. For example, untreated epilepsy can lead to health complications, unaddressed suicide risk can result in fatalities, and unmanaged drug dependence can cause overdoses and severe withdrawal syndromes which can be fatal [15,16]. Additionally, failing to manage mental illness impeded COVID-19 recovery, as individuals with mental health issues struggled to work and reintegrate into their communities, perpetuating poor mental health and socio-economic instability [17]. The lack of appropriate mental health services during the pandemic also led to relapses in previously stable patients [18].

Recognizing the importance of mental health during emergencies, WHO emphasized the inclusion of mental health and psychosocial support (MHPSS) in the COVID-19 response [19]. In Uganda, the MHPSS sub-pillar played a vital role in identifying mental health issues, designing intervention strategies, supporting responder's well-being, supporting those affected by COVID-19, advocating for continued mental healthcare, establishing guidelines, and conducting research [20]. Some of the strategies put in place included training and deployment of mental health staff in COVID-19 treatment units, mental health support for frontline workers, and community engagement meetings to identify mental health needs [20]. However, this was without challenges as one study conducted among undergraduate students in Uganda reported that 51.7% of the participants required emotional support during the pandemic but only 18.3% assessed care from a mental health professional [9].

This study aimed to explore the challenges faced by mental health services and the adaptations of mental health services in Uganda from the perspectives of mental health service users, providers, and policymakers. The adaptations are the strategies implemented specifically in response to the challenges posed by COVID-19 on mental health and mental health services, ensuring the continuity of these services. This information can improve our understanding of the perceived extent of disruptions on mental health services, the reasons for these disruptions, and the mitigation strategies to maintain services.

## Methods

**Study design:** this was a descriptive cross-sectional qualitative study to provide insight into the challenges and adaptations of mental health services during the COVID-19 pandemic.

**Study sites:** participants were recruited from four hospitals: Butabika National Mental Referral Hospital, Gulu Regional Referral Hospital, Hoima Regional Referral Hospital, and Mbale Regional Referral Hospital. Butabika Hospital provides both inpatient and outpatient services for central Uganda and receives referrals from other hospitals. The regional referral hospitals provide inpatient and outpatient services. Additionally, participants were recruited from the mental health office of the Ministry of Health, which oversees MHPSS services nationwide, and from the mental health offices of four districts (Kasese, Masaka, Kitgum, and Tororo), each led by a designated mental health person.

In response to the COVID-19 pandemic, lower-level health facility health workers were trained to screen, identify, and manage mild cases, and to refer severe COVID-19 cases to referral hospitals [21]. The National and Regional Referral Hospitals were equipped with trained health staff, COVID-19 treatment units, and additional supplies of medicines and personal protective equipment (PPE) to manage severe cases. In the regional referral hospitals, mental health units were turned into COVID-19 treatment units as, in most cases, the mental health units were located far from the main hospital making them perfect for isolation of patients with COVID-19 [20].

**Study participants:** we purposively sampled mental health service users, mental health workers, health facility administrators, district mental health focal persons, and mental health policymakers. The service users had actively used mental health services during the COVID-19 period while the health workers provided these services. The administrators, focal persons and policymakers were involved in the coordination of mental health services at hospitals, district, and national levels respectively. The service users were patients receiving treatment at a given facility. The mental health workers had to be psychiatrists, psychiatric clinical officers, or mental health nurses. Health facility administrators had to be members of the administration of the selected hospital. District mental health focal persons are mental health workers who coordinate MHPSS services in the districts and are responsible for coordinating the COVID-19-related MHPSS response at the district level. Mental health policymakers were recruited from the mental health office of the Ministry of Health. All the participants must have been occupying their respective positions for at least six months before the start of the COVID-19 restrictions in Uganda.

**Study tools:** we collected data using a demographic questionnaire and a semi-structured interview. The demographic data included age, sex, education level, and duration of working in each position or receiving treatment from the facility. The interviews explored the experience of provision or utilization of mental health services during the pandemic.

**Data collection:** from April to June 2022, the first author and four research assistants conducted in-person semi-structured interviews individually with participants. Before data collection, the study's purpose was explained, and informed consent was obtained. Interviews with mental health staff, district focal persons, mental health policymakers, and facility administrators were conducted in English, whereas those with service users were conducted in their respective local languages; Luganda for those from Butabika Hospital, Acholi for those from Gulu Hospital, Gishu for those from Mbale Hospital, and Runyoro for those from Hoima Hospital. The interviews were audio recorded using tape recorders with the participant's permission.

**Data management and analysis:** the audio recordings were transcribed verbatim by the data collection team. To ensure transcription accuracy, a second individual fluent in the language reviewed the transcripts. Transcripts in local languages were translated into English by a bilingual speaker fluent in the given local language and English.

Data analysis was conducted by two co-authors using the thematic analysis method. The data analysis involved data exploration for codes and themes were informed by the objectives of the study, that is, challenges faced by mental health services and adaptations of mental health services from the perspectives of mental health service users, providers, and policymakers. Two co-authors read every transcript to become acquainted with the content. They identified significant texts, and assigned codes guided by the study's objectives and prior literature review. They subsequently collaborated to compare, contrast, and consolidate their respective codebooks into a group codebook. The codes were then organized into coherent themes and subthemes. Multiple rounds of review and refinement were undertaken to ensure the presence of coherent patterns. The final themes and subthemes, including verbatim quotations from participants, are presented in the results section.

We employed two forms of triangulation to enhance the validity of our findings. We applied data source triangulation by collecting information from individuals across various cadres, ensuring diverse perspectives. At the analysis level, researcher triangulation was employed, with two researchers initially conducting analyses independently and subsequently comparing notes to derive codes, subthemes, and themes.

**Ethical considerations:** we obtained approval from the Mildmay Uganda Research and Ethics Committee (MUREC-2021-51) and the Uganda National Council for Science and Technology (HS2064ES). Informed consent was obtained from all participants.

## Results

Of the 22 participants enrolled in this study, there were eight mental health service users, four mental health workers, four mental health focal persons, four hospital administrators and two mental health policymakers. The demographic characteristics are represented in Table 1. Themes from the data were organized around the two objectives of the study, that is, the perceived challenges and adaptations of the mental health services. Regarding challenges, the themes were human resource shortages, resource allocation and funding, disruption of patient access to services, and fluctuations in patient load. For adaptations, themes were mobile and remote communication methods, facility adaptations and patient management, change in medication delivery, integration of mental health services into general health services, and alternative financing for mental health services. Table 2 summarizes the themes.

**Table 1:** demographic characteristics of study participants

Variable	Overall n (%)	Mental health service users n (%)	Mental health staff n (%)	Mental health focal persons n (%)	Hospital administration n (%)	Policymakers n (%)
**Sex (frequency)**						
Male	13(59.1)	5(62.5)	2(50.0)	3(75.0)	2(50.0)	1(50.0)
Female	9(40.9)	3(37.5)	2(50.0)	1(25.0)	2(50.0)	1(50.0)
**Age**						
Mean(sd)	43.7(8.0)	42.0(10.2)	45.3(8.4)	48.5(5.9)	42.8(5.7)	40.0(7.1)
**Level of education**						
Primary	3(13.6)	3(37.5)	0(0.0)	0(0.0)	0(0.0)	0(0.0)
Secondary	3(13.6)	3(37.5)	0(0.0)	0(0.0)	0(0.0)	0(0.0)
Tertiary	16(72.7)	2(25.0)	4 (100.0)	4(100.0)	4(100.0)	2(100.0)
**Duration as a patient at a facility or in current work position (years)**						
< 5	6(27.3)	2(25.0)	1(25.0)	1(25.0)	1(25.0)	1(50.0)
5 – 9	7(31.8)	1(12.5)	0(0.0)	3(75.0)	3(75.0)	0(0.0)
10+	9(40.9)	5(62.5)	3(75.0)	0(0.0)	0(0.0)	1(50.0)

Sd: Standard deviation

**Table 2:** summary of themes

Category	Themes
Challenges	Human resource shortages resource allocation and funding disruption of patient access to mental health services fluctuations in patient load
Adaptations	Mobile and remote communication methods changes in medication delivery integration of mental health services into general services alternative financing for mental health services

### Challenges

**Theme 1: human resource shortages**

Participants reported that the pandemic caused significant staff shortages. One of the causes of this was that some staff contracted COVID-19, which prevented them from working.

*"Some of our staff got infected and could not come to work. We also lost one staff to COVID-19. There was also a lot of discrimination of staff that had contracted COVID-19."*- Administrator, Butabika Hospital

Restrictions on public transport during the pandemic prevented some health workers from accessing health facilities. Moreover, police enforced curfew measures that hindered some staff commuting to and from work.

*"Some staff, who were coming from outside the district were stuck there due to the lockdown. The lockdown started with them at their homes, and they were unable to get the hospital for work."* Health worker, Butabika Hospital

*"In the beginning, the police officers never recognized the health workers who were coming to hospital. Some staff were stopped on their way and told to go back home."* Administrator, Hoima Hospital

**Theme 2: resource allocation and funding**

Many mental health units were repurposed for COVID-19 treatment. Mental health teams adapted by offering outpatient services in unconventional locations, such as outdoor spaces. Inpatient services were suspended in some facilities, while others could only accommodate a few patients on non-mental health wards.

*"…the mental health clinic was transferred from its previous location to another location. The initial location was transformed into a COVID unit… The new location was a tent where everyone sits in one place. We didn't have the privacy to share our problems comfortably with the clinician."* Service user, Hoima Hospital

*"One of our wards was improvised for COVID-19…. The patients who were severely sick were referred and those who had moderate illness would stay around on the general ward with other non-mental health patients…"* Focal person, Tororo District

Some participants also reported inadequate funding as a barrier to the delivery of mental health services.

*"We did not get enough money as the MHPSS pillar whereas our colleagues in other pillars managed to get funds for their activities. We had several activities to do, for example counselling for those that had been discharged from COVID-19 treatment units and escorting them back into the communities. Despite all that, somehow, it (the money) wasn't coming through."* Policymaker 2, Ministry of Health

Some participants reported a reduced supply of drugs during the COVID-19 pandemic, with drug stockouts becoming a common occurrence.

*"It was now more common for us to find that there was no medication at the facility. We would be asked to buy the medicine on our own from private pharmacies."* Service user, Hoima Hospital

*"There were also drug stock outs because we focused so much on COVID 19 and almost all the money in the ministry of health budget was channeled to COVID 19. That means that the money to procure medicines for other illness was including mental illness was not sufficient…."* Policy maker 1, Ministry of Health

**Theme 3: disruption of patient access to mental health services**

Lack of access to mental health services was mainly attributed to COVID-19 travel restrictions. Even after the lockdown was lifted, high transport costs continued to discourage some patients from attending clinic visits.

*"…the government instituted a lockdown for three months. Since public transport was banned, we spent three months without getting any treatment…. After the lockdown was eased, the transport costs were high. For a distance where the transport cost was 5,000 shillings, it had doubled to 10,000 shillings…"* Service user, Hoima Hospital

**Theme 4: fluctuations in patient load**

Health facilities experienced fluctuations in patient numbers during the pandemic, initially dropping due to transport restrictions, then sharply rising after restrictions eased.

*"…we had been getting a smaller number of patients coming during the lockdown. But now, the number of patients has increased. …we also have patients who had defaulted treatment during that period, and we have a lot of relapses."* Focal person, Tororo District

### Adaptations

**Theme 1: mobile and remote communication methods**

Participants highlighted the innovative use of mobile and remote communication methods to deliver mental health services during the pandemic. These methods were crucial in overcoming the challenges posed by COVID-19 restrictions and ensuring continued patient care. Mental health workers used mobile phones to maintain contact with patients, providing follow-ups and medical advice remotely.

*"…we had some people that we needed to follow up on and we would call them if we had airtime. We also had some who relapsed because these people were not able to reach us. For those who would reach us by phone, we could offer medical advice. We would direct them to a nearer health facility. If there was any nearby drug shop, we would ask them to buy their drugs from there."* Focal person, Kasese District

The Ministry of Health set up call centers to provide remote mental health support. Patients experiencing mental distress could access counseling services and receive guidance on where to find nearby health facilities.

*"…there were some toll-free lines that patients with mental ill health could call and be supported remotely. If someone was feeling stressed, mental health services like counseling could be provided remotely without someone necessarily coming in. They could get guidance, at least, on where they can get nearby services."* Policymaker, Ministry of Health.

Mental health workers conducted home visits to provide services directly to patients who could not access health facilities, despite the lack of government funding for this approach.

*"…we did home visits. We would go directly to the home and see the patients from home when there was a challenge. This one, however, is not provided for by the government."* Focal person, Masaka District.

**Theme 2: Facility adaptations and patient management**

Health facilities adapted their operations to continue providing mental health services despite COVID-19 disruptions. These adjustments ensured that services remained available and effective. During the pandemic, mental health units were converted into COVID-19 treatment units. Health facilities created new spaces for mental health services.

*"…when our mental health unit was taken up by the COVID-19 services, the health workers strived to make sure that they continued to provide services. They continued to see patients on the veranda instead of sending them back home."* Service user, Gulu Hospital

*"When they removed the mental ward from where the services were, the hospital had to improvise. We had a structure which was renovated where we shifted the mental health services specifically for outpatients."* Administrator, Mbale Hospital

One of the ways to prevent the spread of COVID-19 was through the reduction of overcrowding. Facilities reduced overcrowding by designating specific days for different diseases or age groups, which helped manage workloads and improve service delivery.

*"We had to fix clinic days for specific conditions during COVID-19 and it really helped. Patients started keeping their clinic days. This made work less. It also reduced overcrowding at the clinic…"* Health worker, Hoima Hospital

To further reduce overcrowding, some facilities discharged inpatients early and transport assistance due to public transportation bans.

*"We got some support from partners like 'You belong' in Butabika hospital. They helped in resettling patients who were better to prevent the long stay. …the threshold for discharge was lowered and that for admission was raised."* Policymaker, Ministry of Health

To address transport challenges faced by health workers, health facilities provided free transport or accommodation for staff who lived far away from the health facilities.

*"One of the adaptations was transport of staff and housing of staff during the COVID-19 pandemic. The staff who were staying far from the hospital were given nearby houses."* Administrator, Butabika Hospital

**Theme 3: changes in medication delivery**

To ensure uninterrupted access to medication, health workers extended refill durations and encouraged patients to refill medications at nearby health facilities. Health workers extended medication refills from the usual 30 to 90 days up to 180 days to reduce the need for frequent visits.

*"…we started giving our clients medicine for at least three months. …For those who had not experienced any episodes recently, we would give them drugs for six months."* Focal person, Kitgum District

Patients were also encouraged to refill their prescriptions at nearby health facilities instead of moving up to the regional or national referral hospital ensuring continued access to necessary medications.

*"We would refer some mild cases to lower health facilities, where there is a psychiatric nurse and psychiatric drugs."* Health worker, Mbale Hospital

**Theme 4: integration of mental health services into general services**

Due to increased demand for mental health services, non-specialist health workers received training in basic mental health and psychosocial support (MHPSS).

*"…we trained general health workers in almost all of the regional referral hospitals about mental health so that they could identify a patient in distress and support them or refer them."* Policymaker, Ministry of Health

**Theme 5: alternative financing for mental health services**

In response to government funding shortages, mental health services sought additional financial support from partner organizations and established drug banks. Facilities received financial support from organizations like UNICEF and Butabika Hospital to enhance service delivery.

*"…we received financial support from Butabika Hospital and UNICEF. We would easily reach many people because we had support."* Focal person, Kasese District

Patients set up drug banks, often with partner support, to ensure a steady supply of medications.

*"We also encouraged drug banks. If the patients saved some money, they could buy drugs. They worked with the nearby health centers to keep the drugs so that even if the drugs were not here, they were able to have their own drugs managed by themselves, with the guidance of the health workers. They also got support for this from some partners."* Health worker, Gulu Hospital

## Discussion

This study explores the challenges and adaptations of mental health services in Uganda during COVID-19. The challenges included disruption of service provision and lack of access to mental health services. The adaptations included using innovative methods to deliver mental health services, re-orientating health facility functioning to COVID-19 restrictions, using different methods to provide medications to patients, integrating mental health services in general health services, and alternative financing for mental health services. During the pandemic, there was a shortage of mental health human resources. While these shortages existed before the pandemic [22], they were exacerbated due to staff becoming infected with COVID-19, quarantined, or redeployed within the health system. Similar findings were reported in a multinational study [11]. This resulted in strain on the remaining staff leading to burnout and compromised services [3,23].

Some health facilities in our study converted mental health units into COVID-19 treatment areas, necessitating the creation of alternative spaces for mental health services. While closures of mental health services, particularly inpatient care, for infection control were widely reported [4,14,24,25], transforming into emergency COVID-19 units was less common [12]. These closures or conversions were often due to the healthcare system's unpreparedness, especially in the pandemic's early stages. This is corroborated by a WHO report, which indicated that 93% of surveyed countries experienced disruptions in mental health services [26].

Some participants reported drug shortages in the health facilities. The pandemic exacerbated the pre-existing shortage of psychotropic medications at health facilities in sub-Saharan Africa [27]. Similar issues were reported in South America and were linked to reduced funding and reduced access and distribution of drugs to inpatient and outpatient facilities [28]. To address this, drug banks were established, supported by NGOs, allowing communities to stock up on medications at community health facilities to be accessed during shortages in government facilities. This approach has previously been helpful for patients with epilepsy in Uganda who often face drug shortages [29].

There were changes in the patient numbers during the pandemic, with an initial decline followed by a rise. This pattern of fluctuation in patient numbers has been documented in other settings in the USA [30]. The decline was linked to reduced help-seeking due to fear of infection, government lockdowns and strict hospital policies which deterred patients from accessing health services [11,31,32]. The subsequent increases followed the easing of lockdown measures and were driven by COVID-19 mental health issues and restrictions [3].

Mental health workers used innovative methods like mobile phones and home visits to provide services during the COVID-19 pandemic. Mobile phones are considered a crucial tool in bridging the mental health treatment gap, particularly in low-income and middle-income countries where mobile telecommunications have grown significantly [33]. In Uganda, about 70% of households have a mobile phone [34], making it an accessible means to reach patients. Helplines were also established to provide 24-hour assistance to those in mental distress, as seen in India [13] and Bosnia and Herzegovina [35]. Home visits became an alternative to hospital visits, especially for patients requiring face-to-face evaluation and treatment. Studies in Brazil [36] and the USA [22] noted increased home visits as an alternative to hospitalization for patients with mild-to-moderate symptoms.

To maintain mental health services, health facilities made changes to adapt to the COVID-19 restrictive measures. One of these measures was early discharge and resettlement of patients. Early discharges were used to reduce crowding and, subsequently, COVID-19 infections in inpatient facilities in the UK [23]. Additionally, facilities arranged transportation and accommodations for staff to ensure continued operations during the pandemic, a strategy similarly adopted in HIV care in Uganda [37].

To address rising mental health demands, non-specialist staff were trained and deployed to deliver basic mental health services. This strategy, known as task shifting, had been employed many settings before with promising results [38]. Task shifting allows mental health specialists to attend to more serious cases and facilitates the provision of mental health services to those lacking access to specialists [39].

**Limitations:** in this study, we collected data from various stakeholders, including mental health service users, mental health workers, hospital administrators, mental health focal people and policymakers. However, the mental health service users were recruited from the health facility, potentially excluding those who may still be having challenges with access to mental health services.

## Conclusion

The COVID-19 pandemic created significant challenges for mental health services, primarily due to health system unpreparedness and pandemic-related restrictions. Nevertheless, the health system responded by implementing measures to ensure continuity of care, such as the use of alternative means to reach patients like mobile phones and home visits, multi-month drug dispensation, adjusting clinic operations, and task shifting. Mental health and psychosocial support are vital in epidemic and pandemic responses, underscoring the need for health systems to equip themselves to address emergent mental health challenges, while also ensuring ongoing support for those with pre-existing mental health conditions. Further evaluation of the measures used to support mental health services during the COVID-19 pandemic is recommended to identify opportunities for improvement. Studies can be conducted to examine their impact on mental health service provision and their potential in the post-COVID-19 era.

### What is known about this topic

- *The COVID-19 pandemic significantly impacted mental health services, with increased mental health distress and closure or reduction in services;*
- *Health systems responded to these challenges by reorganizing services to accommodate COVID-19 related changes*.

### What this study adds

- *Changes in mental health services in a low-income setting like Uganda reflect those seen in other settings, though, some were more severe due to already strained health systems;*
- *Despite severity of the challenges and limited health resources in Uganda, there innovative approaches, including relocation of mental health services, task shifting and reliance on mobile phone technology*.
